# A Protein Environment-Modulated Energy Dissipation Channel in LHCII Antenna Complex

**DOI:** 10.1016/j.isci.2020.101430

**Published:** 2020-08-02

**Authors:** Francesco Saccon, Milan Durchan, David Bína, Christopher D.P. Duffy, Alexander V. Ruban, Tomáš Polívka

**Affiliations:** 1 Queen Mary University of London, School of Biological and Chemical Sciences, Mile End Road, E1 4NS London, UK; 2 University of South Bohemia, Institute of Physics, Faculty of Science, České Budějovice, Czech Republic; 3 University of South Bohemia, Institute of Chemistry, Faculty of Science, České Budějovice, Czech Republic; 4 Czech Academy of Sciences, Biology Centre, Institute of Plant Molecular Biology, České Budějovice, Czech Republic

**Keywords:** Physical Optics, Molecular Structure, Optical Property

## Abstract

The major light-harvesting complex of photosystem II (LHCII) is the main contributor to sunlight energy harvesting in plants. The flexible design of LHCII underlies a photoprotective mechanism whereby this complex switches to a dissipative state in response to high light stress, allowing the rapid dissipation of excess excitation energy (non-photochemical quenching, NPQ). In this work, we locked single LHCII trimers in a quenched conformation after immobilization of the complexes in polyacrylamide gels to impede protein interactions. A comparison of their pigment excited-state dynamics with quenched LHCII aggregates in buffer revealed the presence of a new spectral band at 515 nm arising after chlorophyll excitation. This is suggested to be the signature of a carotenoid excited state, linked to the quenching of chlorophyll singlet excited states. Our data highlight the marked sensitivity of pigment excited-state dynamics in LHCII to structural changes induced by the environment.

## Highlights

• Quenched and unquenched LHCII were studied in gel under aggregation-free conditions
• Ultrafast spectroscopy revealed a new spectral band at 515 nm
• The amplitude of the band correlates with quenching in LHCII trimers
• The new band is due to carotenoid-Chl interaction modulated by LHCII conformation

## Graphical Abstract

**Figure:**
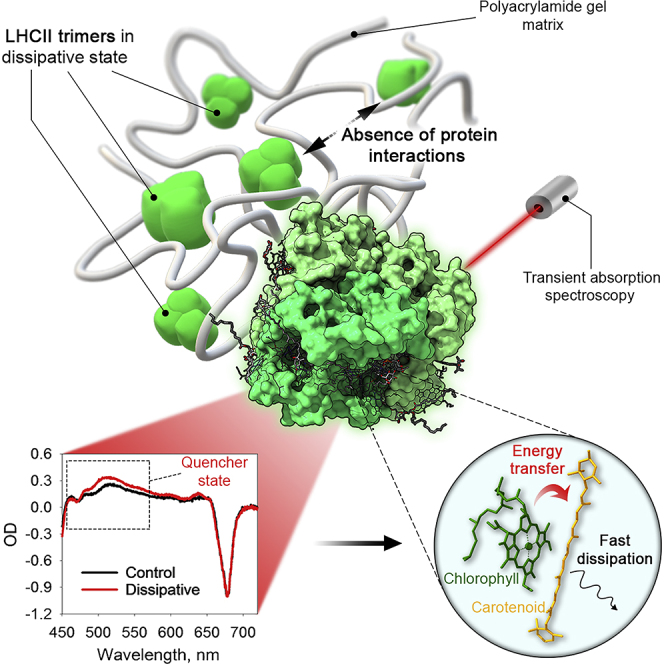

## Introduction

Photosynthesis relentlessly fuels energy to our biosphere, capturing photons of sunlight and storing their energy into stable chemical bonds. Under high light, the photosynthetic apparatus in plant thylakoid membranes is exposed to strong excitation energy pressures, where the harvested number of photons exceeds the capacity to drive photochemistry. To prevent oxidative damage, a diversity of light-harvesting antenna complexes (LHCs), across the eukaryotic photosynthetic lineages, have evolved a photoprotective role and catalyze the release of the excessive excitation energy harmlessly as heat, in a physiological process named non-photochemical quenching (NPQ) (Giovagnetti and Ruban, 2018; Niyogi and Truong, 2013). The mechanism of energy dissipation in higher plants is still debated (Cupellini et al., 2020; Mascoli et al., 2019; Park et al., 2018; Pawlak et al., 2020; Son et al., 2020). Carotenoids (Car) have been proposed to play an essential role in quenching of excess energy, in virtue of their low-lying, short-lived S_1_ excited state (Frank and Cogdell, 1996; Polívka and Sundström, 2004). In several independent studies, the population of Car excited states after direct Chl excitation in LHCs was recorded (Ahn et al., 2008; Avenson et al., 2008; Bode et al., 2009; Holt et al., 2005; Ma et al., 2003; Park et al., 2017, 2018; Ruban et al., 2007). Due to the different dynamical and spectral properties of the obtained signals, various models of quenching mechanisms that include Car participation have been proposed: incoherent slow energy transfer from Chl to Car (Ruban et al., 2007), dissipative excitonic interactions (Bode et al., 2009; Ma et al., 2003; Park et al., 2018), and reductive mechanisms involving electron transfer (Ahn et al., 2008; Avenson et al., 2008; Dall’Osto et al., 2017; Park et al., 2017). The proposal of the former mechanism, first documented in aggregates of major LHCII trimers, has been strengthened by the observation of the same quenching pathway in various systems. It has been reported in cyanobacterial Hlips, ancestral members of the LHC family (Hontani et al., 2018; Niedzwiedzki et al., 2016; Staleva et al., 2015); in CP29 (Mascoli et al., 2019); and in LHCII embedded in membrane nanodisks (Son et al., 2020). Most of these studies provide evidence of the Car S_1_ state to be the quencher, yet the spectrum of the quencher reported recently for CP29 suggests the involvement of another Car excited state, S∗ (Mascoli et al., 2019).

Similarly, the site of NPQ is still a controversial issue, although it is acknowledged that the greater fraction of quenching occurs in the major LHCII trimers, the most abundant pigment-binding complexes in the photosynthetic membrane (Dall’Osto et al., 2017; Nicol et al., 2019; Saccon et al., 2020; Townsend et al., 2018). Each monomer binds 8 chlorophylls a (Chl-a), 6 chlorophylls b (Chl-b), and 4 Car, namely, 2 luteins (Lut), 1 neoxanthin (Neo), and 1 violaxanthin (Vio). Car in LHCII fulfills several functions, related to light harvesting (Gradinaru et al., 2000; Son et al., 2020), structural stability of the trimer (Dall’Osto et al., 2006), Chl triplet quenching (Di Valentin et al., 2009), and Chl singlet quenching (Demmig-Adams, 1990; Ruban et al., 2007).

The flexible design of LHCII allows the complex to switch transiently between unquenched and quenched conformational states (Krüger et al., 2010). The equilibrium between these conformational forms is controlled by environmental cues underlying the reorganization in the thylakoids occurring during the NPQ induction *in vivo*, i.e., pH, Car composition, and aggregation state (Ilioaia et al., 2008; Krüger et al., 2012). Small changes in Chl-Car coupling values, induced by subtle structural changes, can lead to great variations of the excited states' lifetime creating or exacerbating dissipative channels (van Oort et al., 2007). Therefore, LHCII is highly sensitive to its environment and can display a wide range of pigment excited state lifetimes and dynamics (Ilioaia et al., 2008; Moya et al., 2001; Pascal et al., 2005; Ruban et al., 2007; Son et al., 2020).

In this work, we applied transient absorption to LHCII stabilized in the unquenched or quenched conformational state, to investigate the nature of the dissipative switch and the quenching mechanism. The ultrafast spectroscopy techniques exploited to study pigment excited state dynamics are often flawed by singlet-singlet annihilation artifacts. Multi-chromophoric systems such as the light-harvesting thylakoid membranes of plants or LHCII aggregates have been proved to be susceptible to such issues (Müller et al., 2010; van Oort et al., 2018). Therefore, to overcome this problem, isolated LHCII were immobilized in polyacrylamide gels to minimize protein interactions and avoid annihilation artifacts. A comparison with the LHCII trimers and aggregates in buffer revealed the presence of a new spectral band at 515 nm appearing exclusively in gels after Chl excitation. The band is assigned to a Car excited-state absorption and is possibly related to quenching of Chl excitation energy.

## Results

**Figure 1:**
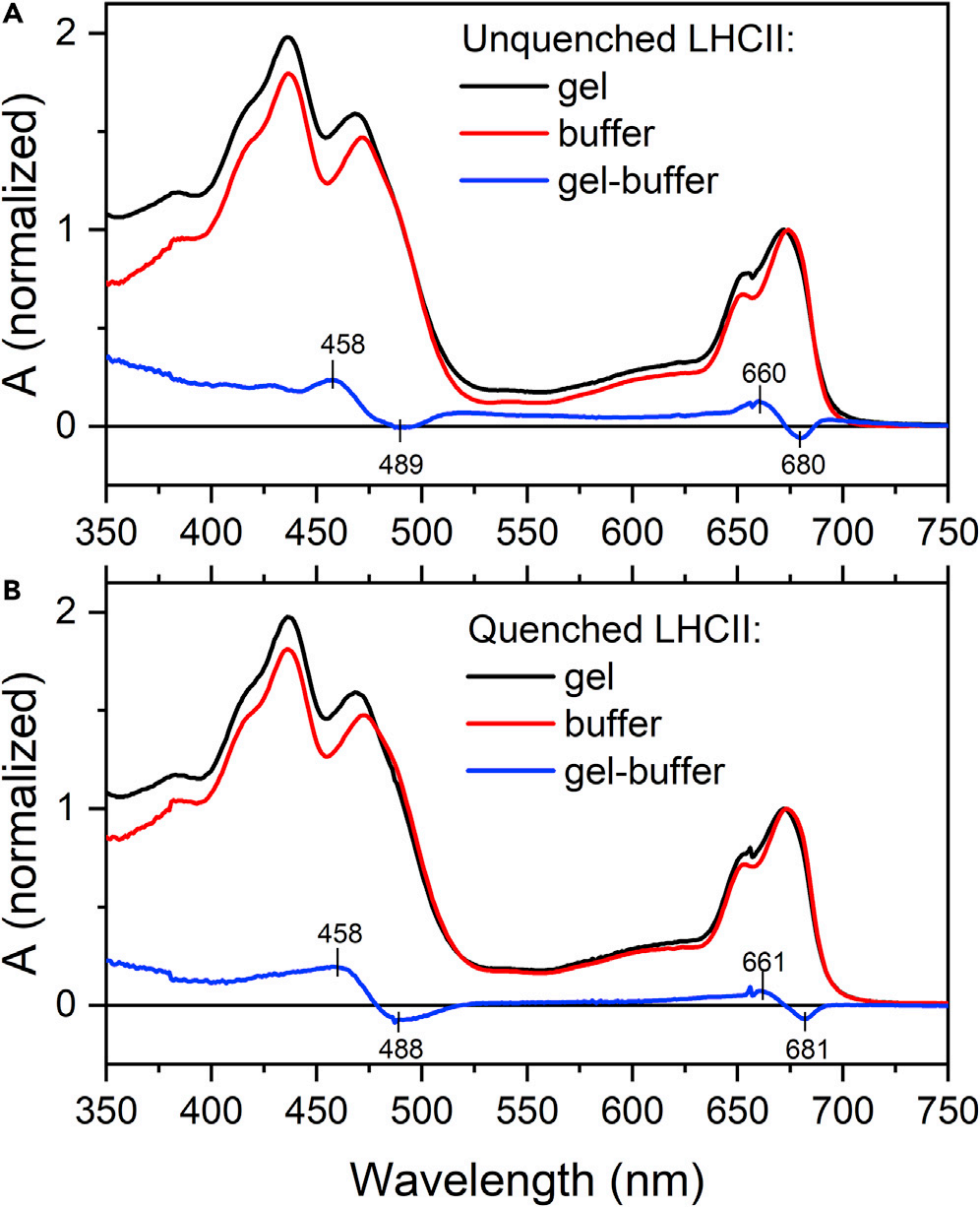
Normalized Absorption Spectra of LHCII Trimers in Buffer and Gel (A and B) (A) Unquenched and (B) quenched LHCII. Spectra are normalized at the maximum of the red absorption band (~675 nm). The difference absorption spectra (gel-minus-buffer) are shown in blue.

Absorption spectra of LHCII trimers in buffer and gel are shown in Figure 1. The comparison shows that immobilizing the LHCII complexes in gel induces some changes as evidenced by the differential spectrum also shown in Figure 1. In the spectral region of Q_y_ band of Chl-a, the immobilization of LHCII in gel causes a 2-nm blue shift of the Q_y_ maximum, which peaks at 674 nm in buffer and at 672 nm in gel. From the differential spectrum, it is obvious that maximal loss of absorption is at 680 nm, whereas maximal gain is at 660 nm, thus some changes in pigment-protein interaction affecting the Chl-a molecules absorbing at these wavelengths are expected. Similar blue shift upon switching from buffer to gel is observed in the Car region where mild absorption loss at 488 nm is compensated by gain at 458 nm. The increased absorption of LHCII gel sample especially at wavelengths shorter than 500 nm is mostly due to an increased scattering in gel, which is much more pronounced in the blue part of the spectrum due to the λ^−4^ dependence of scattering intensity. The changes in absorption spectra induced by immobilizing LHCII in gel are independent of whether LHCII is in quenched or unquenched state. The stabilization of the dissipative conformation leads to subtle differences in the absorption spectra, suggesting that LHCII undergoes minimal structural alterations upon induction of the quenching state (Figure S1).

**Figure 2:**
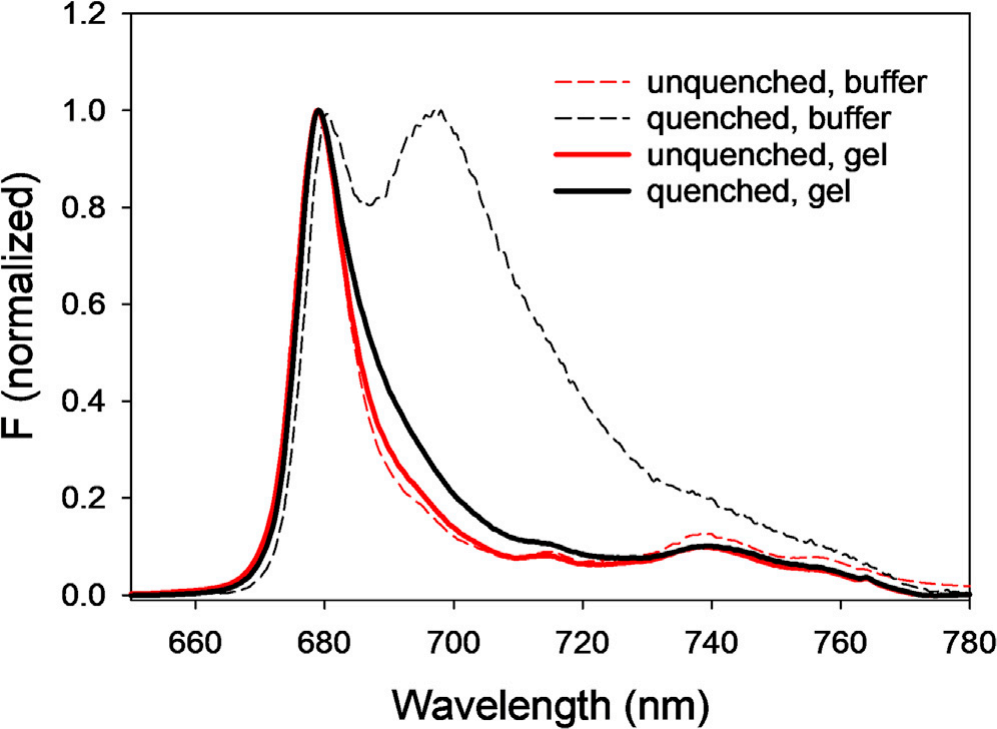
Representative Low-Temperature (77 K) Fluorescence Spectra of LHCII Trimers Data for quenched (red) and unquenched (black) LHCII. Solid lines, LHCII in gel; dashed lines, LHCII in buffer. Spectra are normalized at the maximum of the main fluorescence peak (~680 nm).

The low-temperature fluorescence profile of LHCII immobilized in gels shows a red shift of the main peak at ∼680 nm and spectral broadening toward the red region of the spectrum (Figure 2, solid lines). In contrast to steady-state absorption (Figure S1), the differences between the fluorescence spectra of quenched trimers in gel and quenched aggregates in buffer are pronounced (Figure 2, dashed lines). Upon aggregation in buffer, a very clear emission peak at ∼700 nm appears, which is not visible in quenched trimers in gels. This so-called F700 emission band originates from the formation of Chl charge transfer states, and its relative magnitude often increases during quenching induction (Chmeliov et al., 2019, 2016). It exhibits a good correlation, although not exclusive (Pascal et al., 2005), with the formation of LHCII aggregates (Natali et al., 2016). This marked contrast between fluorescence profiles clearly underlines the differences associated with the preparation of the two samples and the induction of the quenched state. It also constitutes an evidence that immobilization of LHCII trimers in gel prevents aggregation upon quenching induction, as previously shown in detail (Ilioaia et al., 2008).

**Figure 3:**
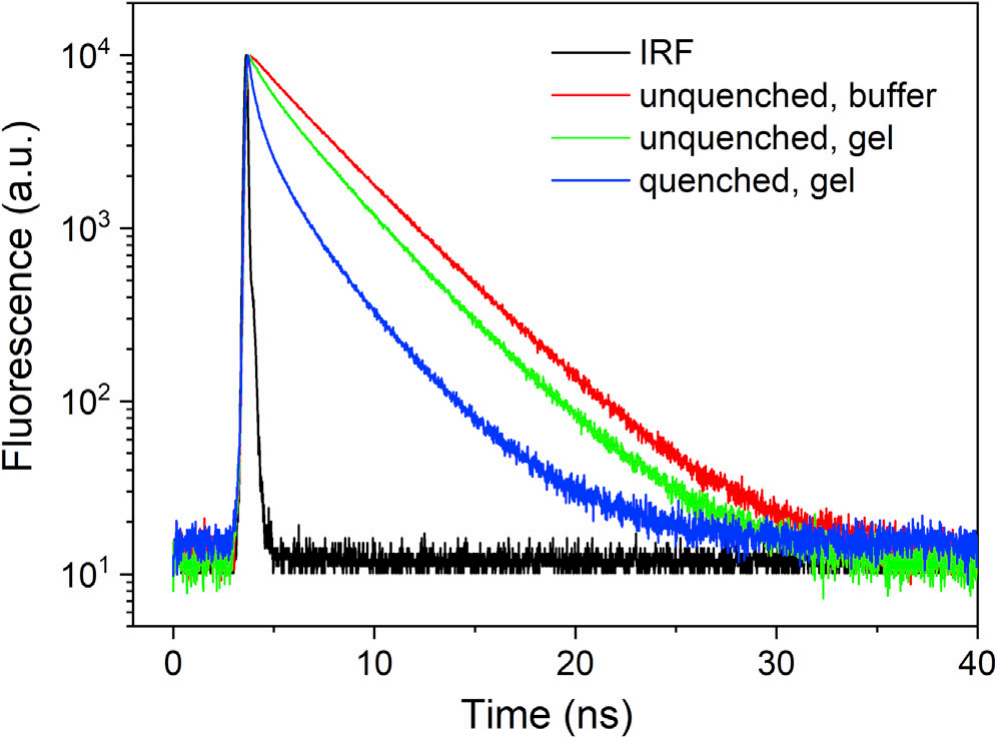
Fluorescence Lifetime Decays of LHCII Trimers Data for unquenched (green) and quenched (blue) LHCII trimers in gels. Excitation was provided at 468 nm, and the fluorescence emission was detected at 680 nm. The fluorescence decay of LHCII trimer in buffer (red) is shown for comparison.

The fluorescence decays of the LHCII trimers in gel shown in Figure 3 clearly prove that immobilization of LHCII in gel does not prevent quenching as the average lifetime of 2.07 ns (see Table 1) of the quenched LHCII in gel is substantially shorter than 3.18 ns obtained for the unquenched LHCII in gel. The average fluorescence lifetime of the unquenched LHCII in buffer, 3.65 ns, is even longer, suggesting that even some mild quenching is introduced when the LHCII trimer is embedded in gel, as previously shown (Akhtar et al., 2015; Rutkauskas et al., 2012). We note that the average lifetime of unquenched LHCII in gel is comparable, but slightly shorter than the 3.73 ns reported by Ilioaia and co-workers (Ilioaia et al., 2008). This could be the result of a very small degree of LHCII clustering induced by the higher protein concentration used in our study. The largest fraction of quenching, however, is achieved after protein immobilization, which prevents further interactions and thus results from structural effects at the level of single trimers. In buffer, all decay components are longer than in gel (Table 1). The major difference between quenched and unquenched LHCII in gel is in the shortest, ∼0.25-ns component, which significantly gains amplitude upon induction of quenching.

**Table 1:** Fitted Decay Times and Relative Amplitudes of LHCII Trimers in Buffer and in Gels

**LHCII in Buffer: Unquenched**						

*τ1 (ns)*	*A1 (rel)*	*τ2 (ns)*	*A2 (rel)*	*τ3 (ns)*	*A3 (rel)*	*<τ> (ns)*
0.57	0.13 ± 0.01	2.96	0.37 ± 0.02	4.11	0.62 ± 0.03	3.65 ± 0.03

**LHCII in Gels: Unquenched**						

*τ1 (ns)*	*A1 (rel)*	*τ2 (ns)*	*A2 (rel)*	*τ3 (ns)*	*A3 (rel)*	*<τ> (ns)*
0.28	0.03 ± 0.01	1.75	0.22 ± 0.03	3.7	0.76 ± 0.03	3.18 ± 0.03

**LHCII in Gels: Quenched**						

*τ1 (ns)*	*A1 (rel)*	*τ2 (ns)*	*A2 (rel)*	*τ3 (ns)*	*A3 (rel)*	*<τ> (ns)*
0.23	0.18 ± 0.01	1.19	0.32 ± 0.01	3.3	0.5 ± 0.02	2.07 ± 0.01

τ*i*, *i*th fluorescence lifetime component; A*i*, fractional amplitude of the *i*th fluorescence lifetime component; *<τ>,* intensity-weighted average lifetimes.

Data are averages of 4 independent measurements ±SD.

**Figure 4:**
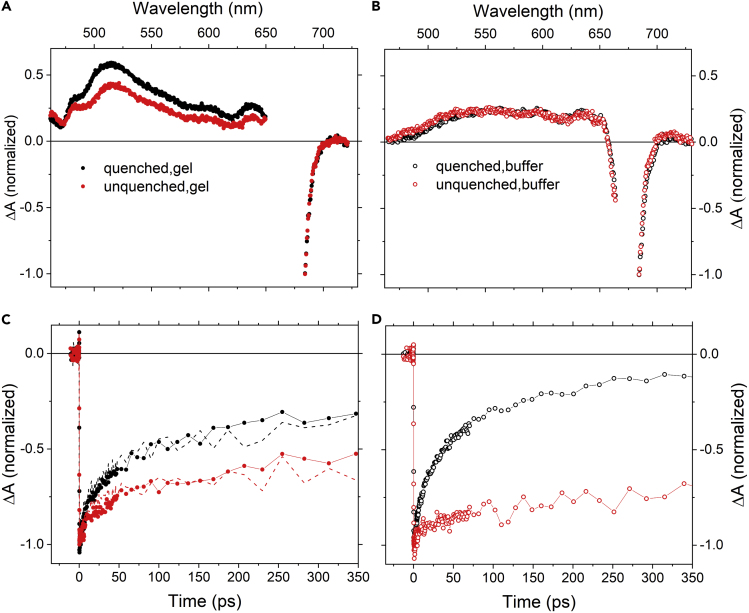
Transient Absorption Profile of LHCII Trimers in Gel and Buffer after Excitation of Chl-a at 674 nm (A and B) Transient absorption spectra of LHCII trimers in gel and buffer, respectively, taken at 10 ps after excitation. The LHCII complexes immobilized in gels are shown as filled dots for quenched (black) or unquenched (red) state. Open symbols with the same color code represent LHCII in buffer. Data from 650 to 680 nm in (A) and from 660 to 680 nm in (B) are omitted due to spoiling of the laser scattering. (C and D) Kinetics monitoring the decay of Chl-a at 684 nm for LHCII in gel (C) and buffer (D), respectively. Color and symbol code are the same in all panels. Data are normalized at 684 nm. The dashed lines in (C) show normalized and reverted kinetics at 515 nm for quenched (black) and unquenched (red) LHCII in gel.

To assess the dynamics in the ultrafast regime, we have applied ultrafast transient absorption spectroscopy. All LHCII samples were excited into the Q_y_ band of Chl-a at 674 nm, and the resulting transient absorption spectra are shown in Figure 4. Only minor changes induced by immobilization in gels are observed in ground-state absorption spectra, whereas properties of excited states are affected significantly. Transient absorption spectra of LHCII immobilized in gel exhibit a new excited state absorption band peaking at 515 nm (Figure 4A), which is missing when LHCII trimers are resuspended in a buffer (Figure 4B). Moreover, whereas the spectral profile of transient absorption spectrum of LHCII in buffer is the same for quenched and unquenched sample, in gel the quenched sample exhibits a larger amplitude of the 515 nm band (related to the amount of excited Chl-a as the spectra in Figure 4 are normalized at 684 nm) than the unquenched sample.

Data in Figure 4 suggest that the 515 nm band may have a direct relation to the quenching capacity of LHCII trimers, but kinetics shown in Figures 4C and 4D reveal a more complicated picture. The decay parameters of the excited Chl-a clearly do not correlate with magnitude of the 515 nm band. Quenched LHCII in buffer exhibits the shortest Q_y_ lifetime, but does not show the presence of the 515 nm band at all (Figure 4B). Thus, the appearance of this band is not a necessary condition to observe quenching, although it is clearly a result of embedding the LHCII trimers in gel. LHCII in buffer exhibit significantly different dynamic properties of quenched and unquenched samples, yet spectral profiles of their transient absorption spectra remain essentially identical.

**Figure 5:**
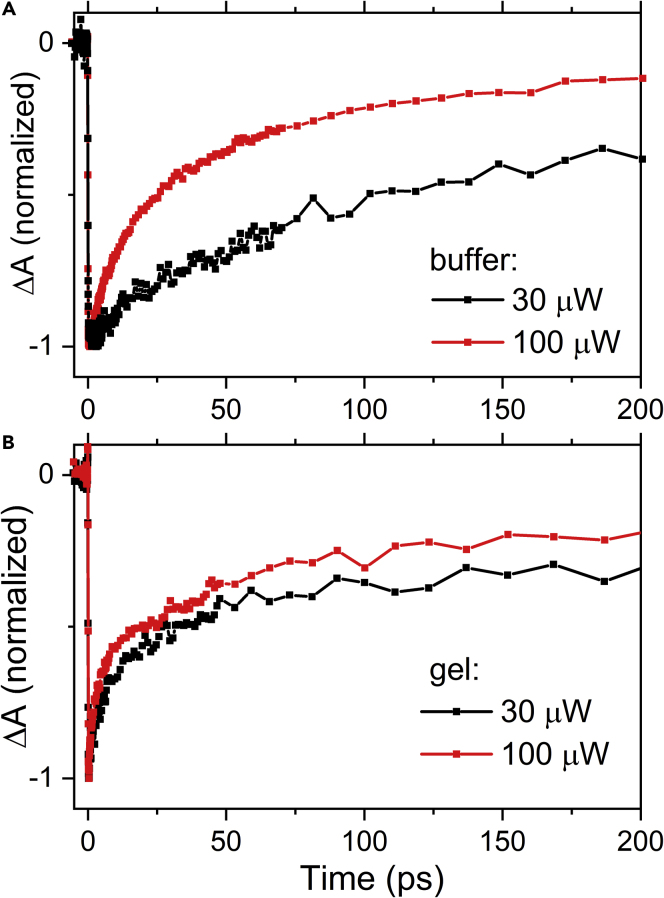
Absorption Kinetics Measured at 685 nm for Different Excitation Intensities (A and B) (A) Quenched LHCII in buffer and (B) quenched LHCII immobilized in gel. Excitation was provided at 674 nm.

The immobilization of LHCII complexes in gel prevents protein-protein aggregation, which inevitably arises in LHCII resuspended in buffer when decreasing the detergent below the critical micellar concentration. Data shown in Figure 4 demonstrate that quenched LHCII complexes immobilized in gel have a visibly shorter Chl-a lifetime after quenching induction. As the LHCII complexes in immobilized gel form only a negligible amount of aggregates, the observed quenching originates from an intrinsic conformational change occurring in isolated trimers (Ilioaia et al., 2008). As the data for quenched and unquenched LHCII complexes were measured with the same sample concentration and excitation intensity, the difference in decay properties cannot be due to annihilation. If annihilation is present, it must be the same in both quenched and unquenched complexes in gel. Yet, the large change in Chl-a lifetime in quenched and unquenched LHCII complexes in buffer can be partly caused by a higher extent of annihilation in quenched aggregates (van Oort et al., 2018). To check this, we carried out experiments with different excitation intensities for quenched LHCII complexes in gel and in buffer (Figure 5). The data demonstrate that there is indeed almost no annihilation in LHCII complexes immobilized in gels, whereas LHCII in buffer clearly contains a significant annihilation component. This confirms that the shorter Chl-a lifetime in LHCII in gel must be related to processes occurring in individual LHCII trimers. Furthermore, the absence of annihilation also suggests that the 515 nm band cannot be generated via the re-pumping mechanism suggested by van Oort et al. (van Oort et al., 2018) who showed that a Car-like band can be generated from upper Chl-a states populated via annihilation.

What is then the origin of the 515 nm band? We show in Figure 4C that decay of the 515 nm band correlates with decay of Chl-a in the Q_y_ bleaching region. Normalized and reverted kinetics taken at the maximum of the 515 nm band overlay nicely with the decay of Q_y_ band at 684 nm, demonstrating identical decay properties of the 515 nm and Chl-a Q_y_ bands. This may invoke a conclusion that the 515 nm band is due to an excited-state absorption of Chl-a, but some arguments are rather against such assignment. Any Chl excited-state absorption signal reported in the 500–560 nm spectral region displays a lack of structure and defined features and exhibits a low magnitude. In this region of the spectrum, instead, the absorption signal of Car excited states is predominant (Polívka and Sundström, 2009). In fact, upon closer inspection of the 460–520 nm spectral region, the blue part of the spectrum exhibits a structure reminiscent of bleaching bands superimposed on a broad positive background. Thus, Car may also be suspected to be responsible for the presence of the 515 nm band. The position of the weak bleaching band at 490 nm reasonably matches the expected absorption bands of lutein in the L1 site of LHCII (Ruban et al., 2000). Furthermore, a similar spectral band has been recently reported for CP29 and assigned to the S∗ state of lutein (Mascoli et al., 2019). We note, however, that the 515 nm band reported here has a much larger amplitude and is also broader than the S∗ band reported by Mascoli et al. (Mascoli et al., 2019).

**Figure 6:**
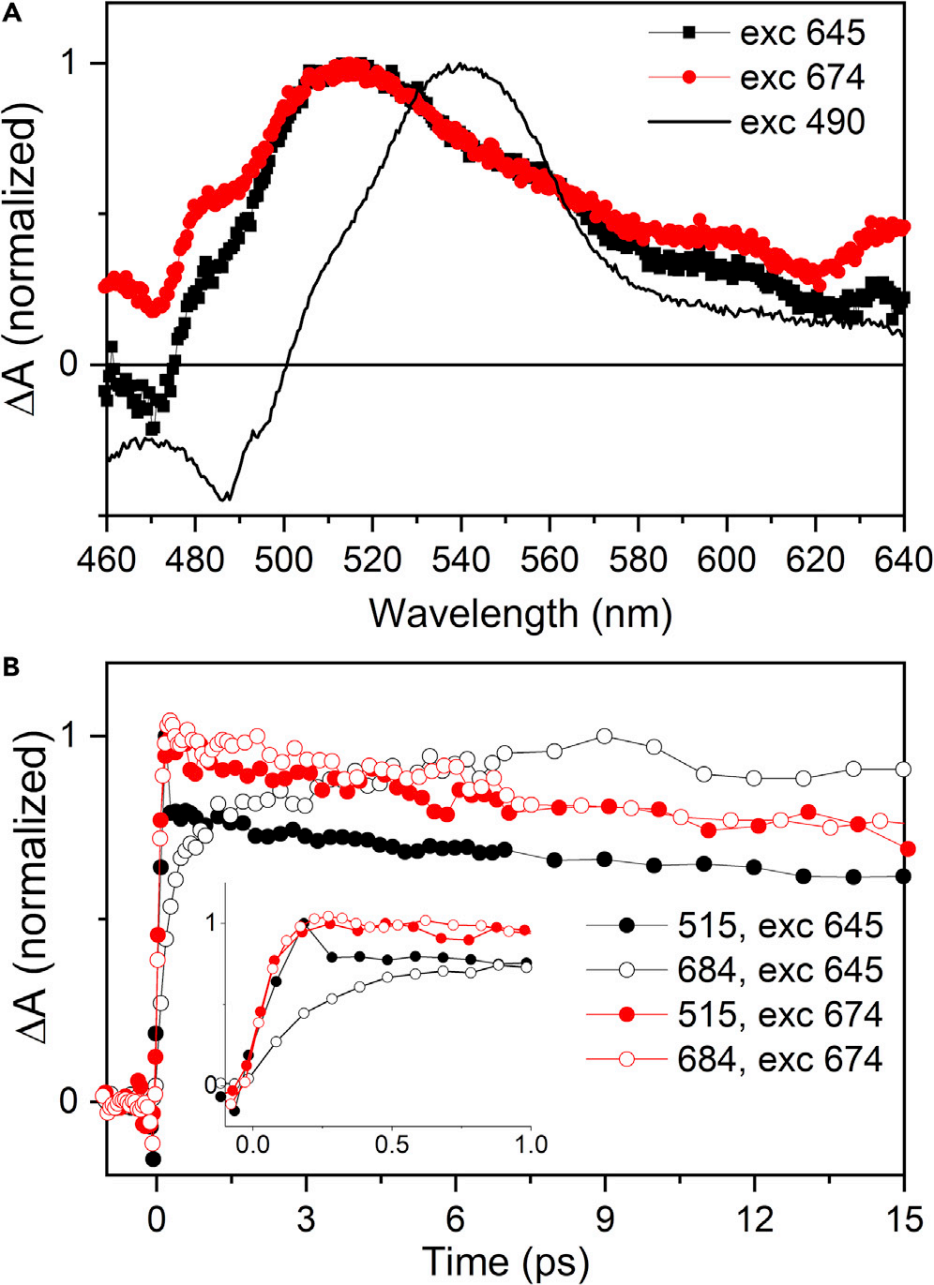
Spectroscopic Properties of the 515 nm Band in Quenched LHCII Trimers in Gel (A) Spectral profiles of the 515 nm band 10 ps after excitation of Chl-a at 674 nm (red) and Chl-b at 645 nm (black). For comparison, the S_1_-S_n_ of carotenoid excited at 490 nm (black line) is also shown. (B) Kinetics showing the instantaneous rise of the 515 nm band (full symbols) after excitation at 674 nm (red) and 645 nm (black). Reverted kinetics of Chl-a bleaching (open symbols) after 674 and 645 nm excitations are shown for comparison. Inset enlarges the first picosecond. All data are normalized to maximum.

The assignment of the 515 nm band to the lutein in L1 could invoke Chl-a quenching via energy transfer to the S_1_ state of lutein reported earlier for LHCII (Ruban et al., 2007) and also other systems (Park et al., 2018; Pinnola et al., 2016; Staleva et al., 2015). However, data depicted in Figure 6 suggest a more complex scenario. To show spectral and dynamical properties of the 515 nm band we focused on the quenched LHCII sample in gel that has the strongest 515-nm signal. In Figure 6A we compared the Car spectral region of transient absorption spectra measured after excitation of Chl-a, Chl-b, and Car. Excitation to either Chl-a or Chl-b generates the 515 nm band, whereas direct excitation of Car at 490 nm generates the characteristic S_1_-S_n_ spectrum peaking at 540 nm, preventing assignment of the 515 nm band to a conventional S_1_-S_n_ transition of lutein. Yet, it is worth noting that the blue shoulder, typically denoted as the S∗ signal (Polívka and Sundström, 2009), matches reasonably the maximum of the 515 nm band (Figure 6A). This indicates that the possible energy acceptor cannot be the S_1_ state. Instead, as Mascoli et al. suggested for CP29, the S∗ state of lutein could be involved also in LHCII (Mascoli et al., 2019). Furthermore, we note that at longer delays after excitation at 490 nm, at 100 ps when the S_1_ state of the Car has decayed, the resulting transient absorption spectrum is nearly identical to that measured after direct excitation of Chl-a (see Figure S2). Thus, the 515 nm band appears regardless of whether we excite Chl-a or Car, in the latter case being hidden under the strong S_1_-S_n_ transition at earlier delay times.

Dynamical properties of the 515 nm band are summarized in Figure 6B. Its long lifetime, matching the decay of Chl-a (Figure 4C), does not match the expected lifetime of lutein S_1_ state, and rather suggests the presence of Car-Chl coupling (Bode et al., 2009; Park et al., 2018). The 515 nm band does not exhibit any rise time and appears instantaneously within our time resolution. Interestingly, however, the immediate appearance of the 515 nm band is independent of whether Chl-a or Chl-b is excited (Figure 6B, inset). When Chl-b is excited at 645 nm, the Chl-a bleaching detected at 684 nm exhibits a clear rise, reflecting the dynamics of Chl-b to Chl-a energy transfer, but no such rise is observed at the maximum of the 515 nm band. This observation somehow points to involvement of Chl-b in formation of the 515 nm band, but we note that there is no Chl-b bleaching accompanying the appearance of the 515 nm band (Figure 4). Thus, excitonic interaction between Chl-b and Car is unlikely to be the origin of the 515 nm band.

**Figure 7:**
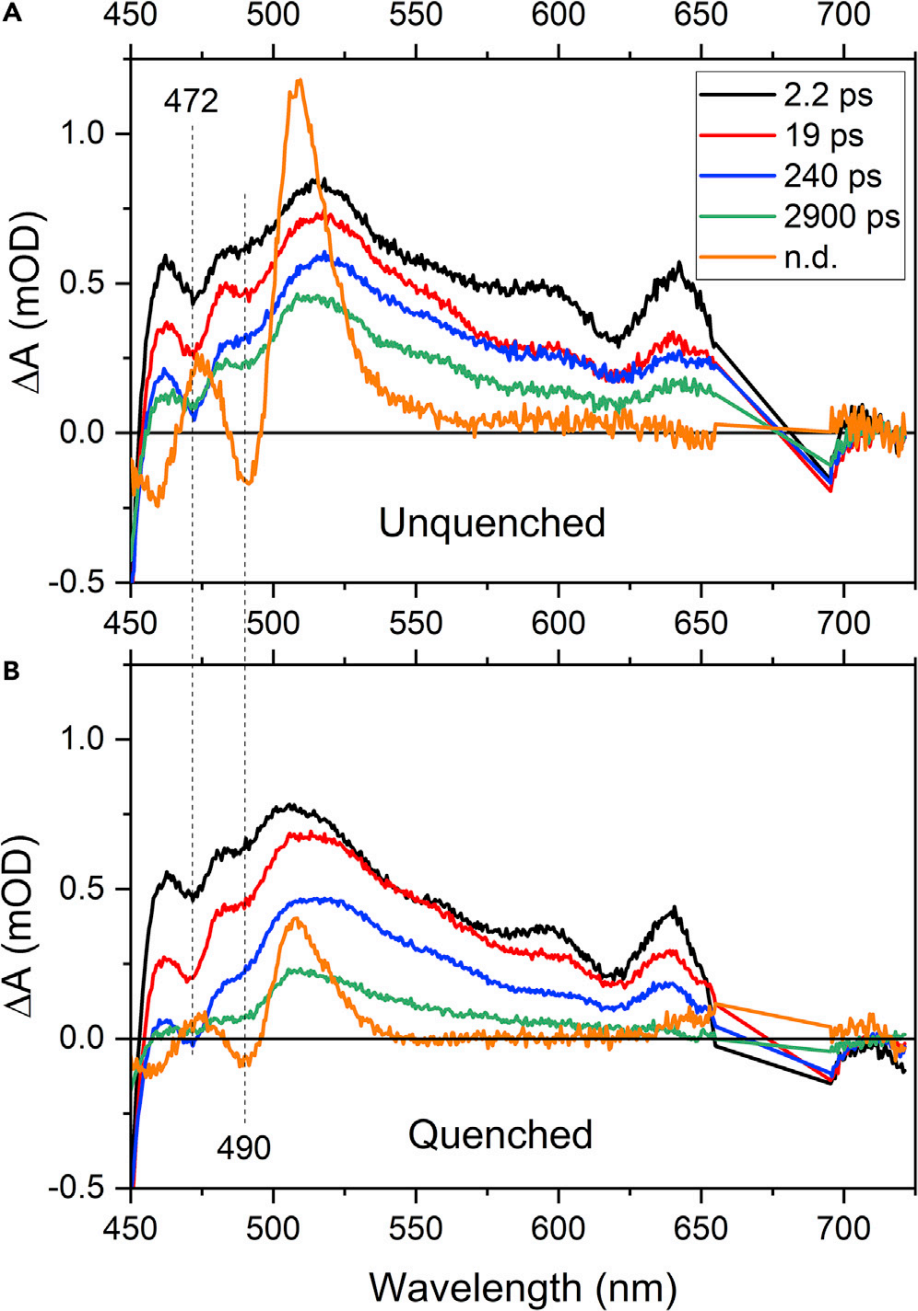
EADS Obtained from Global Fitting of the Data Measured for LHCII in Gel Excited at 674 nm (A and B) (A) Unquenched and (B) quenched LHCII. The two prominent negative bands in the carotenoid bleaching region are denoted by vertical lines. The 650–700 nm spectral region is removed from the fitting due to strong scattering from the 674 nm excitation.

To gain better insight into the dynamics of the 515 nm band, we applied a global fitting procedure to the collected data. We used a sequential model for the data excited either to Chl-a or Chl-b and made use of data obtained from fluorescence decays (Figure 3) to put constraints on the long time components. The results are shown in Figure 7 for LHCII in gel excited at 674 nm. Data collected after Chl-b excitation are presented in Supplemental Information (Figure S4). A minimum of four decay components is needed to fit the LHCII trimers in gel excited at 675 nm. We used the same set of time components for both quenched and unquenched LHCII as the fitting in a number of trials converge to very similar time constants in both samples, differing only in amplitudes. To test whether we do not over-parametrize the fitting, we tested fitting with less time components (Supplemental Information, Figure S5).

The longest component, 2.9 ns, corresponds to the average of the two longest values obtained from fluorescence decays (Table 1), because the time window used for the transient absorption measurements does not justify using a component longer than 3 ns. The averaged value of 2.9 ns captures appropriately the long time dynamics of the system. The 240-ps component matches the fastest component obtained from fluorescence decays. As in the fluorescence decays, the contribution of this component, manifested as a difference between the 240-ps evolution associated difference spectrum (EADS) and the following one (2.9 ns), significantly gains amplitude upon induction of quenching (see also amplitude spectra shown in Figure S3). Similar, but not that pronounced behavior is observed for the 19-ps component, which also has slightly higher amplitude in the quenched LHCII in gel. The fastest, 2.2-ps component is essentially identical in both quenched and unquenched LHCII and has therefore negligible role in quenching. The spectral profiles of the individual time components confirm the immediate appearance of the 515 nm band. The spectral profile of individual EADS does not change much, signaling that all spectral features have very similar decay properties, following the decay of Chl-a excited state. Only the final, non-decaying component has a distinct spectral shape, clearly corresponding to a carotenoid triplet state populated via triplet-triplet energy transfer from Chl-a. The amplitude of the triplet spectrum is markedly weaker in the quenched sample, confirming that quenching prevents population of Chl-a triplet. The data obtained after Chl-b excitation (Figure S4) require one extra component to reproduce the Chl-b to Chl-a energy transfer (0.4 ps), but other time constants are comparable to those obtained with Chl-a excitation. All time constants extracted from global fitting are summarized in Table 2.

**Table 2:** Summary of the Fits of the Transient Absorption Data of the LHCII in Buffer (Unquenched) and the LHCII Embedded in Gels

LHCII in Gel: Quenched and Unquenched					
	*τ1 (ps)*	*τ2 (ps)*	*τ3 (ps)*	*τ4 (ps)*	*τ5 (ps)*
Excitation at 674 nm		2.2 (±0.03)	19 (±0.3)	240 (±4)	2,900 (fixed)
Excitation at 645 nm	0.4 (±0.002)	3 (±0.02)	38 (±0.6)	280 (±6)	2,900 (fixed)

The quenched and unquenched samples were fitted together as described in the text. The numbers in parentheses indicate the bootstrap estimates of the 68% (1σ) confidence intervals of the time constants, obtained by bootstrapping as described in Supplemental Information, Figure S6. The time constants (in ps) are sorted by magnitude to facilitate comparison. Non-decaying component due to a carotenoid triplet observed in all analyzed dataset is omitted.

We note that the time components extracted from our data exhibit some resemblance with those reported by Mascoli et al. who reported components with 2.4, 54, and 2,400 ps lifetimes (Mascoli et al., 2019). Here, however, we also identify the 250-ps decay process, which appears in both fluorescence and transient absorption data, proving that it cannot be related to annihilation. Instead, this process likely represents the major quenching component for LHCII trimers immobilized in gels. Furthermore, in our data, the amplitudes of the components are much larger in the 500–600 nm spectral region than in Mascoli et al. (Mascoli et al., 2019) due to the presence of the 515 nm band. The data obtained after excitation at 645 nm have an additional fast (0.4 ps) component describing the Chl-b to Chl-a energy transfer, but the overall picture is comparable to that obtained for the data excited at 675 nm, including the immediate appearance of the 515 nm band (see Supplemental Information, Figure S4).

## Discussion

The conundrum of the quenching mechanism in LHCII is a long-standing issue, due to the intricate network of proteins and pigments enabling it *in vivo*. The NPQ induction is indeed characterized by the interplay of LHC proteins, the PsbS subunit, and the xanthophyll cycle (Derks et al., 2015). Ultimately, these last two factors are dispensable for the quencher formation in LHCII, although being pivotal in the responsiveness of the complexes to the energization of the membrane during high light exposure (Saccon et al., 2020). To minimize the effect of protein interactions and investigate the conformational switch of single LHCII trimers, we immobilized isolated complexes in polyacrylamide gels, preventing aggregation from occurring during induction of a dissipative state. Despite the possible presence of negligible clustering during the embedding procedure, the greatest extent of quenching occurs after immobilization, via structural changes of single complexes induced by the removal of detergent molecules. Previous studies have shown the remarkable similarities of the LHCII switch in gel with the spectroscopic changes occurring upon NPQ formation in thylakoids (Akhtar et al., 2015; Ilioaia et al., 2011, 2008). These shared features involve spectral broadening of fluorescence with a concomitant red shift and marked absorption changes associated with the lutein near the terminal emitter chlorophyll cluster (Ilioaia et al., 2008). Structural data obtained by resonance Raman spectroscopy confirm the occurrence of distortions of Lut and Neo molecules (Ilioaia et al., 2011), in analogy to changes observed in leaves and thylakoids (Ruban et al., 2007). Circular dichroism data also report a strong perturbation of Neo-specific bands, caused by the extraction of detergent from the gel matrix (Akhtar et al., 2015). Ilioaia et al. (Ilioaia et al., 2011, 2008) identified this as the signal of altered Neo and Lut interactions with other pigments, as evidenced by the Raman spectral changes.

Overall, the commonalities with *in vivo* data legitimize the use of the LHCII gel platform as a means to uncover the signature of the quenched state and bridge it to the recent NPQ research. We must note, however, that LHCII is a congested pigment-protein complex, on the verge of a dissipative state. Quenching, therefore, is likely not an on/off switch, and the gel induction could be only one of several possible quenched configurations in the thylakoids.

Our data show that the immobilization of LHCII in gel generates a spectral feature in the data that have not been reported so far, the 515 nm band in transient absorption spectra. It is tempting to assign the origin of the band to the S∗ of lutein, in line with the recent report by Mascoli et al. who suggested S∗, there assigned to a separate excited state, to be a quencher in CP29 (Mascoli et al., 2019). Yet, there is significant difference between the data reported by Mascoli et al. and the data reported here. In Mascoli et al., the band peaking at 510 nm and assigned to the quencher (S∗ state) is not directly visible in transient absorption spectra and is recreated only after target analysis of the data. This is because the lifetime of the quencher is much shorter (∼6 ps) than the lifetime of the quenched Chl-a (50 ps), thus the actual population of the quencher is extremely low (Mascoli et al., 2019). Here, however, the picture is different as the 515 nm band has an amplitude reaching 50% of the Chl-a bleaching (Figure 4A), preventing application of the model with “reverse kinetics” (the quencher is populated slower than is its lifetime). Our data instead suggest that the state generating the 515 nm band is significantly populated. We also note another distinction between ours and Mascoli's data: whereas in Mascoli et al. the spectrum of the quencher closely matches the spectrum of Car triplet state (Mascoli et al., 2019), in our data the triplet spectrum differs from the spectral profile of the 515 nm band (Figure 7).

Yet, can we still assign the 515 nm band to the S∗ of lutein, even though the mechanism of its formation must be different from Mascoli et al.? The spectral position of the band reasonably matches the expected lutein S∗ spectrum, although the 515 nm band extends further into longer wavelengths. The current view of the origin of the S∗ signal in solution points toward contributions from both a vibrational shoulder of S_1_ and “hot” ground-state effects (Balevičius et al., 2016; Ehlers et al., 2018). The hot ground-state contribution to the S∗ signal is probably a combination of long-lasting vibrational excitation and slow heat dissipation to the environment (Balevičius et al., 2019). Even though a “hot” ground state is the inevitable product of rapid excitation quenching by S_1_, it is clearly not the origin of the 515 nm band. Vibrational excitation of ground state cannot occur faster than the decay of S_1_, whereas the 515-nm signal appears instantaneously and is directly coupled to the Chl-a/b bleach.

Alternatively, it is possible that the 515-nm signal arises from a distinct electronic excited state. As the Chl excited-state absorption band is typically flat and featureless it seems more likely that it is somehow related to a carotenoid excited state, possibly to a specific configuration in the S_1_ state that has also been proposed as one of the origins of the S∗ signal (Gradinaru et al., 2001; Liguori et al., 2017; Mascoli et al., 2019). If this is the case, then the lack of a rise time component implies an extremely fast redistribution of energy from the Chl pool to this S_1_-like state. Recently, Son et al. (2020), observed Chl Q_y_ to Car S_1_ energy transfer occurring on a timescale of ∼300 fs in quenched LHCII, although this is still slower than what we observe here. The appearance of the 515 nm is instantaneous with our ∼100 fs time resolution, which, if it really is a reflection of Chl-Car energy transfer, would require very strong Chl-Car couplings. Interestingly, it was recently argued that strong couplings and fast transfer do not result overall in particularly strong quenching (Balevičius and Duffy, 2020). This is due to an entropic effect in which the strongly coupled Chl-Lut pair gradually receives energy from a much larger Chl pool. This same “drip feeding” effect could also explain the relatively long lifetime of the 515-nm peak, although it does not explain its large amplitude.

**Figure 8:**
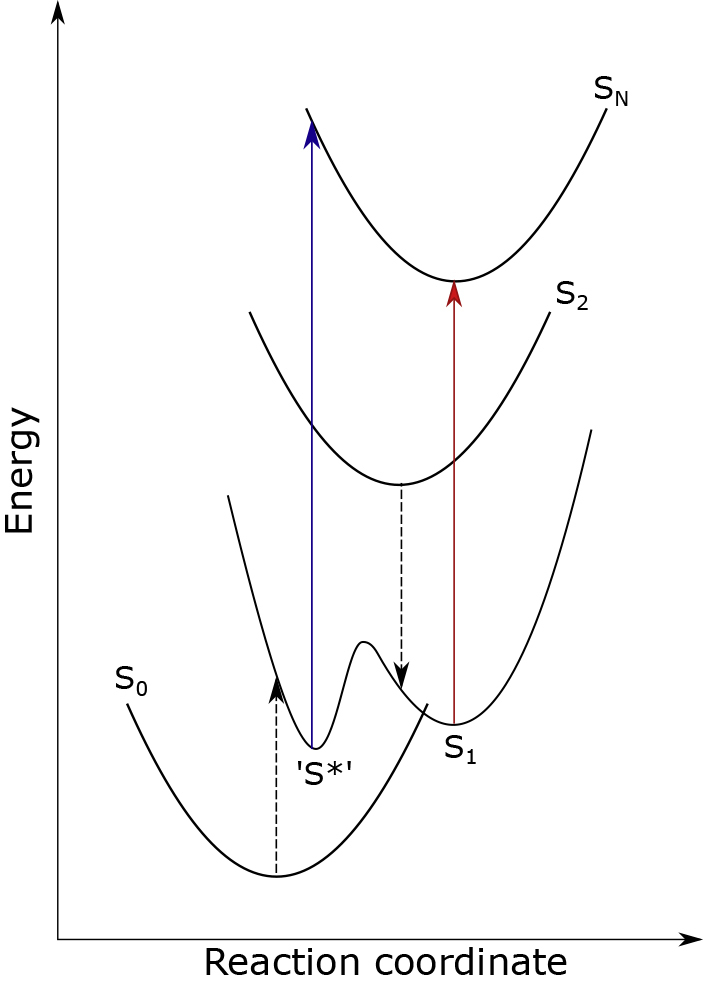
Basic Scheme for the Additional Minimum/State Hypothesis of the 515-nm Signal S_1_ is inaccessible from the ground state but readily accessible via ultra-fast interconversion from S_2_ (downward dashed arrow). Direct excitation of S_2_ leads to the 540-nm excited state absorption peak of S_1_ (red arrow). Transfer of energy from Chl leads to direct excitation of our “S∗” state from the ground state (upward dashed arrow). Depending on the shape and position of S_n_ it is possible that this leads to a slightly blue-shifted ESA (blue arrow). This diagram is purely for illustrative purposes.

Excitonic interactions are often invoked when such fast energy redistribution is observed. The 25 nm difference between the 515-nm peak and the usual position of S_1_ is of the order of what one would expect from excitonic peak shifts, and it could be suggested that the 515-nm peak originates from absorption from a red-shifted, S_1_-like excitonic state. However, one would expect to also see this excitonic peak when exciting the carotenoid directly. Instead, we see the normal 540-nm peak arising from S_1_ absorption, although the 515 nm band appears at later delay times even after Car excitation (Figure S2, Supplemental Information). As noted earlier (Staleva et al., 2015), an explanation may lie in the “direction” from which S_1_ is excited. When populated from the Chl pool, S_1_ is excited electronically from the ground state, whereas direct carotenoid excitation involves interconversion from S_2_, probably via a conical intersection (Hauer et al., 2007; Liebel et al., 2014). Within a simplified displaced harmonic oscillator model, whether S_1_ is populated from the ground state or S_2_ would have no effect on the position of S_1_ absorption. However, for a more complex potential energy surface, one with two or more minima, the absorption position could be sensitive to how this surface is accessed. This idea is sketched in Figure 8. It is important to note that the x axis does not refer to any particular coordinate (such as an end-ring dihedral, for example) but represents some general direction of distortion. Indeed, Ilioaia et al. have shown that during the onset of quenching lutein undergoes some non-trivial twisting to its conjugated backbone (Ilioaia et al., 2011). Although this could in principle be represented as some linear combination of different normal coordinates, here we draw our potential energy surfaces for illustrative purposes only. Essentially, the global minimum of S_1_ is accessible only from S_2_, whereas transition from the ground state accesses either some local S_1_ minimum or another strongly coupled dark state. We note that a comparable explanation of the S∗ signal has been reported earlier for carotenoids in solution, and the distortion generating the S∗ minimum of the S_1_ potential surface was either the torsion angle of the terminal rings (Niedzwiedzki et al., 2006) or small dihedral distortions of the single and double C=C bonds in a linear carotenoid (Niedzwiedzki et al., 2007). If such an additional minimum/state is responsible for the 515-nm peak, then it must only be accessible from the highly distorted ground-state geometry present in LHCII in gel and not for solubilized LHCII. In this model, the 515 nm band is assigned to S∗ as in Mascoli et al. (2019), but the mechanism by which it is populated (and consequently also the quenching mechanism) differs entirely.

However, we must note that our hypothesis of distortions granting access to an otherwise inaccessible state is further complicated by the fact that the 515-nm peak appears instantly when both Chl-a and Chl-b are excited. Chl-b-carotenoid couplings in LHCII are perfectly possible, and they likely involve neoxanthin (Fox et al., 2017), whereas Chl-a couples to lutein. It seems unlikely that these two different carotenoids would both be perturbed in such a specific manner. Yet, the assignment of the 515 nm band to some excited state associated solely with lutein is also not straightforward. The lutein bleaching bands are readily observable in the triplet spectrum (the non-decaying EADS in Figure 7) that is known to originate largely from the triplet of lutein in the L1 site (Mozzo et al., 2008). The bleaching of the 0-0 band of lutein is at 490 nm, which in fact matches the weak negative band appearing along with the 515 nm band (e.g., red EADS in Figure 7 or data shown in Figure 4A). Yet, the triplet EADS in Figure 7 also reveals the 0-1 bleaching band of lutein at 460 nm, yielding the correct ∼1,350 cm^−1^ energy gap between the 0-0 and 0-1 vibronic bands. This is, however, not the case of EADS featuring the 515 nm band, as the main negative band is at 472 nm (Figure 7), implying that the negative bands superimposed on the 515 nm positive bands are not the 0-0 and 0-1 vibrational bands of a single carotenoid. In fact, various analyses of the carotenoid absorption profiles reported earlier did not assign any carotenoid band to 472 nm; the closest would be 0-1 band of the “red” lutein in the L2 site at 476 nm (Ruban et al., 2000). However, such assignment would require 0-0 band around 510 nm, which is undetectable in the data presented here. Nevertheless, the bleaching bands suggest that they do not originate from a single carotenoid, supporting the hypothesis that more than one carotenoid should be involved to explain the appearance of the 515 nm after both Chl-a and Chl-b excitation.

### Conclusions

In conclusion, even though we are not able to unequivocally assign the origin of the 515 nm band that has been induced by immobilization of LHCII in gel, our data provide important insights into the quenching mechanism(s). The data presented here underscore the importance of subtle structural changes in LHCII as the key tuning factor in regulating the flow of energy through LHCII. Immobilization of LHCII in gel probably generates a distribution of structural variants of LHCII having subtle differences in Chl-Car couplings. The quenching induction by removal of the bound detergent molecules then shifts the distribution more toward the quenching structures, simulating the process likely occurring *in vivo* in the natural environment of LHCII, the thylakoid membrane.

Membrane lipids are crucial for the structural stabilization of antenna proteins and their functionality in the native thylakoids (Schaller et al., 2011; Seiwert et al., 2017). The lateral membrane pressure profile, dictated by different lipid types around LHCII, might be pivotal in tuning the fluorescence yield of the complexes (Tietz et al., 2020). The local environment of membrane proteins has also a strong impact on their mobility and controls their tendency to interact with other proteins (Killian, 1998; Quemeneur et al., 2014). Recently, it has been hypothesized that a reorganization of the lipids bound to LHCII is associated with a hydrophobic mismatch around the complexes, and this in turn locks the dissipative state of the antennae in the thylakoid membrane (Daskalakis et al., 2019; Ruban, 2019).

Although the conundrum of the nature of the quenching mechanism in LHCII remains unsolved, due to the technical limitations in studying native systems, our data indicate that the LHCII function is tuned dramatically by changes in the surrounding local environment, and these can be the driving force of its photoprotective switch during NPQ. This is also in line with recent report of ultrafast Chl-Car energy transfer channel in LHCII identified by 2D electronic spectroscopy. Son et al. (2020) showed that this energy transfer channel operates only for a Chl-Car with specific spectral properties as it was observed only for narrow spectral range within the broad S_1_-S_n_ Car band. Our data also show that there could be more than one dissipative pathway involved in photoprotection, reflecting the heterogeneity in the structure of the pigments bound to LHCII and their relative orientation and coupling. LHCII is an intrinsically dissipative system, and, as such, maintaining a light-harvesting state is less trivial than achieving its counterpart, the quenched state. It thus seems that LHCII evolved a flexible structure, enabling it to transiently “switch off” during NPQ, and this process is essentially governed *in vivo* by the interplay between membrane and protein dynamics.

### Limitations of the Study

We have shown here that certain interactions in LHCII complex can generate a new spectroscopic feature in transient absorption spectra whose magnitude correlates with quenching. However, we cannot determine the exact origin of these interactions at atomic level, because we do not know how embedding in gel affects the LHCII structure. As absorption spectra of LHCII gel differ only marginally from those measured in a buffer, it is expected that the appearance of the 515 nm band in transient absorption spectra will depend on small changes in LHCII structure, possibly at the level of a single amino acid. Future experiments should focus on spectroscopic studies of LHCII mutants embedded in gels to monitor the presence or absence of the 515 nm band described in this study to provide more information about possible structural changes induced by embedding LHCII in gel. Such information will allow access to detailed calculations of chlorophyll-carotenoid interactions, eventually testing the validity of the scheme shown in Figure 8. The scheme currently provides a hypothesis, which is feasible yet unsupported by calculations of excited-state properties. Finally, our study is limited to *in vitro* system, and future extension to environments closer to physiological conditions, such as, e.g., ultrafast spectroscopic studies of LHCII in membrane, should provide more information about the NPQ conformation of LHCII. It is possible that the specific LHCII conformation described in this study is one of several that may occur *in vivo*.

### Resource Availability

#### Lead Contact

Further information and requests for resources and reagents should be directed to and will be fulfilled by the Lead Contact, Tomáš Polívka (tpolivka@jcu.cz).

#### Materials Availability

This study did not generate any new materials or reagents.

#### Data and Code Availability

The data reported in this study are available from the Lead Contact on request.

## Methods

All methods can be found in the accompanying Transparent Methods supplemental file.

## Supplemental Information

Document S1. Transparent Methods and Figures S1–S6

## Footnotes

Supplemental Information can be found online at https://doi.org/10.1016/j.isci.2020.101430.
