# Supplementary material for: A Protein Environment-Modulated Energy Dissipation Channel in LHCII Antenna Complex
Source: iScience. 2020 Aug 2;23(9):101430. doi: 10.1016/j.isci.2020.101430 (PMC7452274; doi:10.1016/j.isci.2020.101430)
Supplement: Document S1. Transparent Methods and Figures S1–S6 [file mmc1.pdf]

iScience, Volume *23*

Supplemental Information

A Protein Environment-Modulated

Energy Dissipation Channel

in LHCII Antenna Complex

Francesco Saccon, Milan Durchan, David Bína, Christopher D.P. Duffy, Alexander V. Ruban, and Tomáš Polívka

Supplemental Information

A protein environment-modulated energy dissipation channel in LHCII antenna complex

Francesco Saccon, Milan Durchan, David Bína, Christopher D. P. Duffy, Alexander V. Ruban, and Tomáš Polívka

Supplemental information

Transparent methods

Sample Preparation. Major LHCII trimers were isolated from thylakoids of WT Arabidopsis thaliana via flat-bed preparative iso-electric focusing, as previously described (Dainese and Bassi, 1991; Ruban et al., 1994). Unstacked thylakoids were solubilized with 11.5 mM n-dodecyl-βD-maltoside (βDDM; β-DDM/Chl=20). The trimeric LHCII fraction was collected in buffer containing 25 mM HEPES and 200 μM βDDM, at pH 7.6. To induce the quenched conformation of complexes in buffer, isolated LHCII (100 μg/ml) were resuspended in a glass cuvette in the presence of adsorbent polystyrene BioBeads, under constant stirring, to achieve the gradual removal of detergent from the solution, thereby favoring LHCII aggregation (Ruban et al., 2007). The process was stopped by separation of LHCII fraction from the beads pellet. Immobilization of LHCII into 1 mm thick poly-acrylamide gels was carried out as previously described (Ilioaia et al., 2008; Saccon et al., 2019). The induction of the quenched conformation of LHCII in gels was also achieved by removal of detergent bound to the protein complexes, via overnight incubation of the gels at room temperature in detergent-free buffer (25 mM HEPES, pH 7.6) under constant slow stirring. Immobilization in gels, however, prevented LHCII aggregation, as previously shown (Ilioaia et al., 2008). Both quenching inductions (LHCII in buffer and in gel) were monitored measuring quenching of the Chl-a fluorescence signal using a Dual PAM fluorometer (Walz, Germany), applying a weak measuring blue light (<12 μmol photons m-2s-1) and detecting the fluorescence signal as an integral of wavelength >700 nm. Comparable quenching extents were reached in both treatments.

Steady-State Spectroscopy. All absorption measurements were performed at room temperature. Steady-state absorption spectra were measured on Agilent 8453 UV-VIS diode array spectrophotometer. Fluorescence emission spectra of LHCII in buffer and in gel (10 μg/ml total Chl concentration) were recorded at 77 K using a Jobin Yvon FluoroMax-3 spectrophotometer equipped with a liquid nitrogen cooled cryostat. Excitation was performed at 435 nm with 5 nm slit width and the fluorescence spectral resolution was 0.5 nm. Integration time was set to 0.1 s. Every spectrum is the average of 5 scans.

Time-Resolved Fluorescence Spectroscopy. Time-resolved fluorescence spectroscopy was performed at room temperature using a Fluotime 200 time-correlated single-photon counting setup (Picoquant, Germany). Excitation was provided by a 468 nm laser diode and pulse frequency was set to 20 MHz. The laser power used in the experiment was 0.6 mW, corresponding to ~30 pJ/pulse. Fluorescence was detected at 680 nm with 2 nm of slit width. Measurements of unquenched LHCII in buffer were performed in a glass cuvette (1 cm) at 10 μg/ml total Chl concentration. Isolated LHCII were resuspended in 2 ml of a solution containing 25 mM HEPES, 200 μM βDDM, pH 7.6. LHCII in gels (1 mm thick, 100 μg/ml total Chl concentration) were fitted into a plastic holder and placed in the sample compartment at 45° relative to the excitation and emission slits. Fitting was performed using the software FluoFit (Picoquant, Germany), with a multi-exponential model with iterative re-convolution of the instrument response function (IRF, 50 ps). Average lifetimes were calculated as $\Sigma_i(A_i \cdot \tau_i)/\Sigma_i A_i$, where Ai is the amplitude of i-th lifetime component and τ_i is the respective fluorescence lifetime value.

Transient Absorption Spectroscopy. Transient absorption spectra were measured by a modular laser system consisting of an ultrafast Ti:sapphire regenerative amplifier (Spitfire Ace-100F, Spectra-Physics, USA) seeded with a Ti:sapphire oscillator (MaiTai SP, Spectra-Physics, USA), and pumped by Nd:YLF laser (Empower 30, Spectra-Physics, USA). The laser system produces ~100 fs pulses centered at 800 nm with a 1-kHz repetition rate. The produced pulses were divided into excitation and probe beams by a beam

splitter. Tunable excitation pulses were generated by an optical parametric amplifier (TOPAS-C, Light Conversion, Lithuania). The probe pulses were generated by focusing a fraction of the 800 nm beam to a 2-mm sapphire plate to generate a broadband (450-750 nm) white light pulse. In order to minimize chirp, the white light beam was collimated by an off-axis parabolic mirror and split by a broadband 50/50 beam splitter to a reference and probe beam. The probe beam was focused by a 300 mm spherical mirror to a sample where it overlaps with the excitation beam. Probe and reference beams were then focused to the entrance slit of a spectrograph where the beams were dispersed onto a double CCD detection system (Pascher Instruments, Sweden) allowing measurements of transient spectra in a spectral window of ~250 nm. The time delay between the excitation and probe pulses was introduced by a computer-controlled delay line. The mutual polarization of the excitation and probe beams was set to the magic angle (54.7°) by placing a polarization rotator in the excitation beam. Using neutral-density filters, the excitation intensity in all experiments was kept at ~ 4.0 x 1013 photons pulse-1 cm-2. The LHCII gel sample of a 10 x 10 mm size and of 1 mm thickness was squeezed between two quartz windows with a 1 mm Teflon spacer. The cuvette was placed into a holder attached to a Lissajous 2D scanner that moves the sample during the measurement to prevent degradation. The spectro-temporal datasets were fitted globally. The fitting results are visualized either as evolution associated difference spectra (EADS) resulting from fitting the data to a sequential kinetic scheme, in which individual excited-state species evolves according to a sequential, irreversible scheme, or as decay associated difference spectra (DADS) corresponding to spectral profiles of amplitudes associated with individual time components.

REFERENCES

Dainese, P., Bassi, R., 1991. Subunit stoichiometry of the chloroplast photosystem II antenna system and aggregation state of the component chlorophyll a/b binding proteins. J. Biol. Chem. 266, 8136–8142.

Ilioaia, C., Johnson, M.P., Horton, P., Ruban, A. V., 2008. Induction of Efficient Energy Dissipation in the Isolated Light-harvesting Complex of Photosystem II in the Absence of Protein Aggregation. J. Biol. Chem. 283, 29505–29512.

Ruban, A. V., Berera, R., Ilioaia, C., van Stokkum, I.H.M., Kennis, J.T.M., Pascal, A. a, van Amerongen, H., Robert, B., Horton, P., van Grondelle, R., 2007. Identification of a mechanism of photoprotective energy dissipation in higher plants. Nature 450, 575–578.

Ruban, A. V., Young, A.J., Pascal, A.A., Horton, P., 1994. The Effects of Illumination on the Xanthophyll Composition of the Photosystem II Light-Harvesting Complexes of Spinach Thylakoid Membranes. Plant Physiol. 104, 227–234.

Saccon, F., Durchan, M., Kana, R., Prášil, O., Ruban, A. V., Polívka, T., 2019. Spectroscopic Properties of Violaxanthin and Lutein Triplet States in LHCII are Independent of Carotenoid Composition. J. Phys. Chem. B 123, 9312–9320.

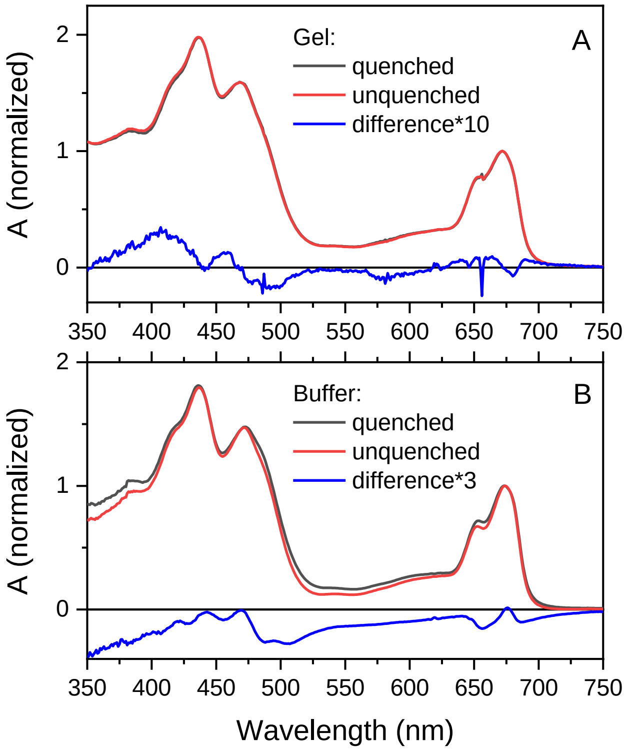

Figure S1. Comparison of absorption spectra of quenched and unquenched LHCII trimers. Related to Figure 1. Absorption spectra of LHCII in gel (A) and buffer (B) in unquenched (red) and quenched (black) state. The blue line shows difference between unquenched and quenched state.

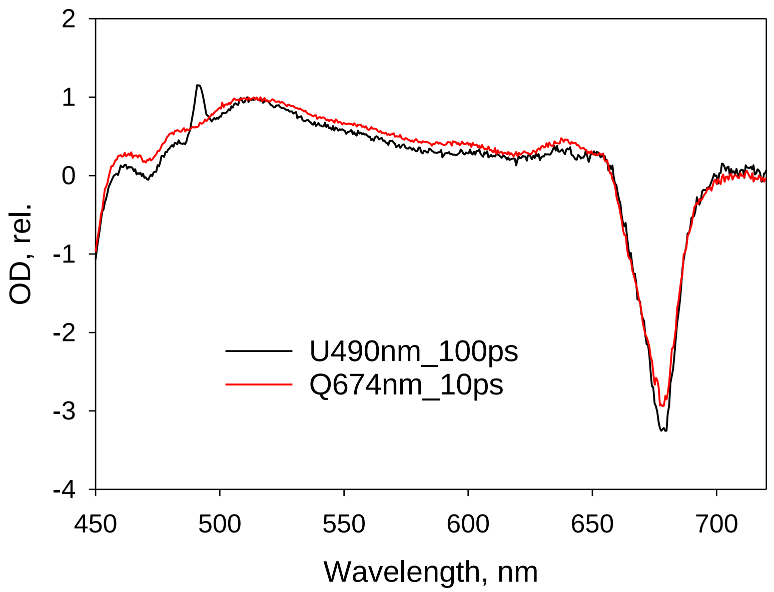

Figure S2. Comparison of transient absorption spectra of LHCII trimers after different excitations. Related to Figure 6. The quenched LHCII in gel were excited at 674 nm (black), unquenched LHCII in gel excited directly to the carotenoid at 490 nm. The spectra are taken at 10 ps (excitation at 674 nm) and 100 ps (excitation at 490 nm). The spectra are normalized at the maximum of the 515 nm band.

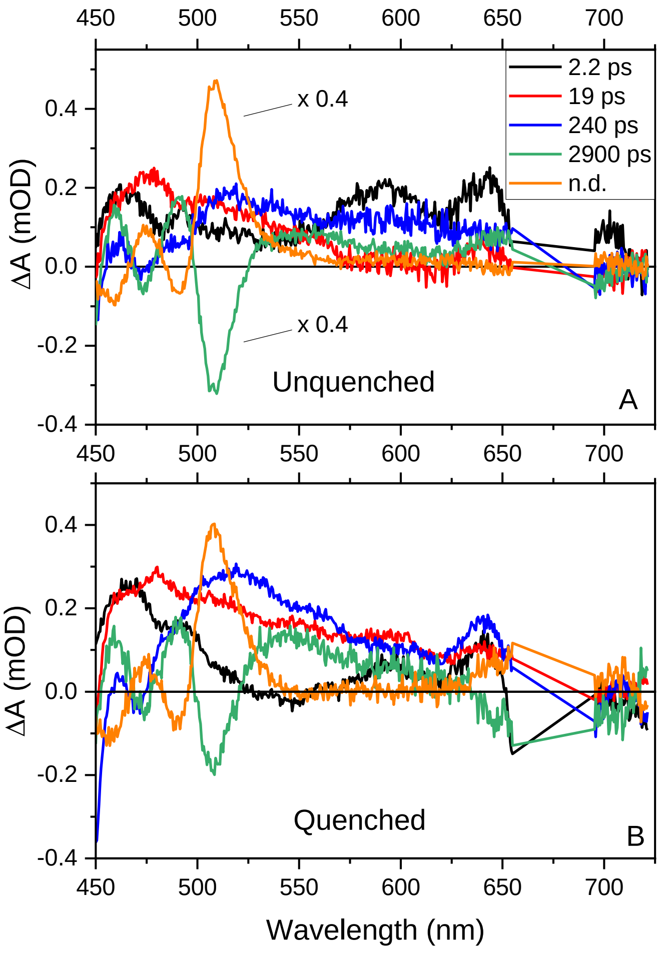

Figure S3. Spectral profiles of amplitudes of time components (decay-associated difference spectra, DADS) obtained from global fitting the data measured for LHCII in gel excited at 674 nm. Related to Figure.7. (A) Unquenched, (B) Quenched LHCII. The 650-700 nm spectral region is removed from the fitting due to strong scattering from the 674 nm excitation. Note that the component associated with triplet rise in the unquenched LHCII has been multiplied by 0.4 to visualize other components.

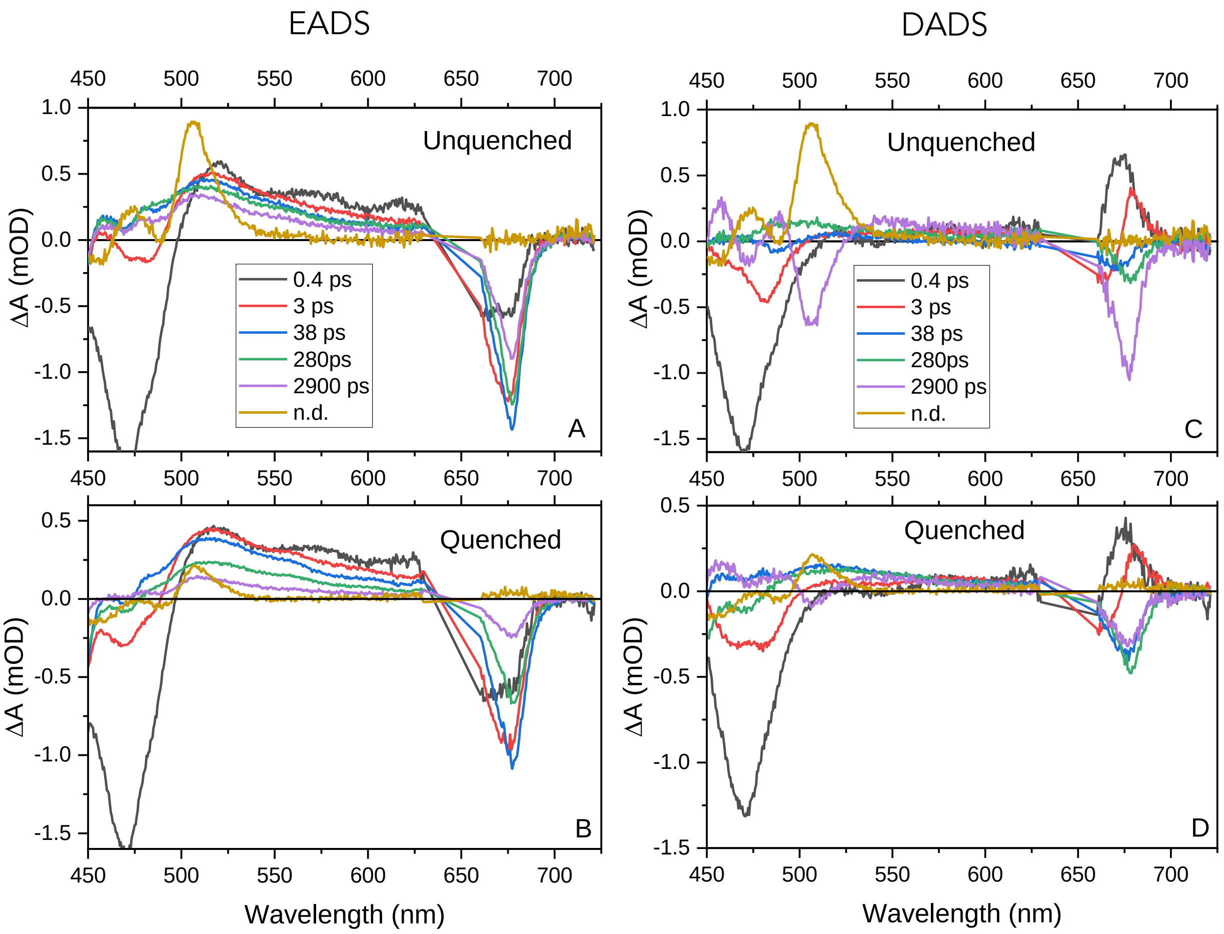

Figure S4. EADS (A, B, left) and DADS (C, D, right) obtained from global fitting the data measured for LHCII in gel excited into Chl-b at 645 nm. Related to Figure.7. (A, C) Unquenched, (B, D) Quenched LHCII. The 630-660 nm spectral region is removed from the fitting due to strong scattering from the 645 nm excitation.

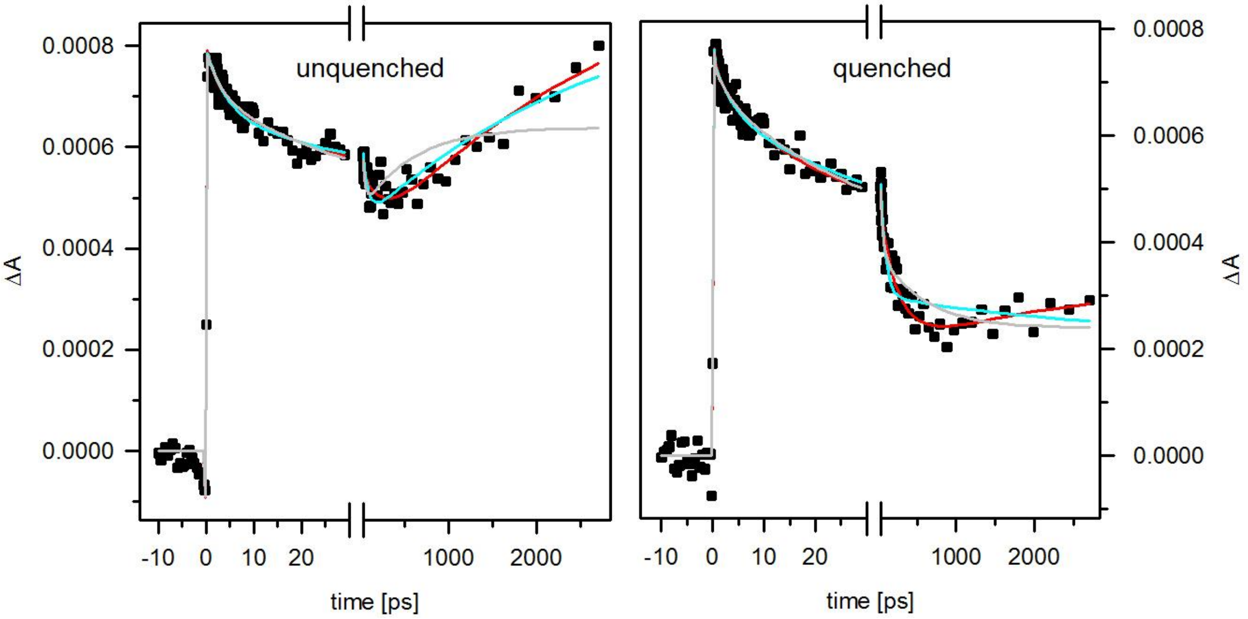

Figure S5. Comparison of the models of excited state dynamics using a different number of kinetic components. Related to Figure 7. Kinetics of absorption changes at 512 nm measured on LHCII trimers in gel fitted by 5-component (4 kinetics + non-decaying triplet, *red*) and 4-component model (3 kinetics + non-decaying triplet, *cyan*). The slowest component was fixed to 2900 ps in both cases. Also shown is the 4-component model with free slow component (*grey*). The quenched and unquenched data were fit together by a single set of rate constants. Corresponding EADS and the time constants of the 5-component model are given in Figure 7. The 5-component model was clearly superior in capturing the 100 ps – ns range of the excited state dynamics, in particular in the quenched sample. Moreover, the 4-component fit yielded the time constants of 4 ps, 63 ps and failed to retrieve the ~200-300 ps component observed in the time-resolved fluorescence (see Table 1). Moreover, the *grey* line shows that fixed slowest component was necessary to capture the dynamics of the carotenoid triplet.

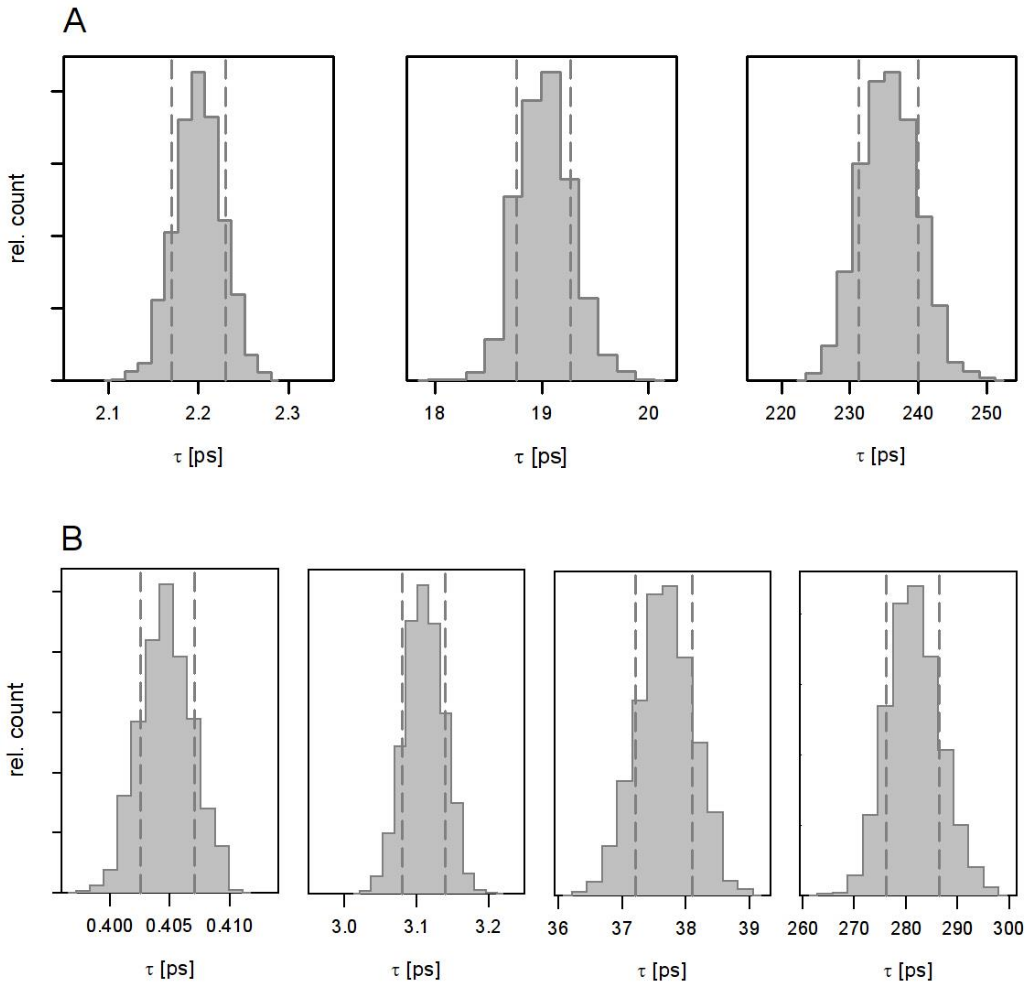

Figure S6. Histograms obtained by bootstrap tests of the time constants of the fits of the transient absorption data of LHCII embedded in gels. Excitation at 674 nm (A) and at 645 nm (B). Related to Figure 7 and Table 2. The quenched and unquenched samples were analyzed together. The analysis was performed as follows: the experimental datasets were fitted with the model, yielding the "model" data (=fit) and residuals (data - model). Then, a new dataset was generated by adding randomized residuals to the model. This was repeated to generate 1000 surrogate datasets that were subsequently fitted, yielding the displayed distributions of time constants that can be used to estimate the confidence intervals (Kuznetsov et al., 2017). Dashed lines indicate 68 % (1σ) confidence intervals.

Reference

Kuznetsov I.A., Kuznetsov A.V. (2017) Using Resampling Residuals for Estimating Confidence Intervals of the Effective Viscosity and Forchheimer Coefficient. Transp. Porous Med. 119, 451-459.
